# What are the attributes of a good health educator?

**DOI:** 10.5116/ijme.5765.0b6a

**Published:** 2016-06-28

**Authors:** Dragan Ilic, Jessica Harding, Christie Allan, Basia Diug

**Affiliations:** 1 Medical Education Research and Quality Unit, School of Public Health and Preventive Medicine, Monash University, Australia

**Keywords:** Health educator, medical education, standards

## Abstract

**Objectives:**

The purpose of this study was to examine the attributes that students and educators believe are important to being a good health educator in a non-clinical setting.

**Methods:**

A cross-sectional survey of first-year health science students and educators involved with a Health Science course in Melbourne, Australia was performed. A convenience sampling approach was implemented, with participants were required to rate the importance of teaching attributes on a previously developed 15-item written questionnaire. Descriptive statistics were generated, with Pearson’s chi-square statistics used to examine differences between groups. In total 94/147 (63.9%) of students and 15/15 (100%) of educators participated in the study.

**Results:**

Of the 15 attributes, only ‘scholarly activity’ was not deemed to be not as an important attribute to define a good educator. Knowledge base (50% vs. 13.3%) and feedback skills (22.3% vs. 0%) were rated as important attributes by students in comparison to educators. Professionalism (20% vs. 5.3%), scholarly activity (20% vs. 3.2%) and role modelling (26.7% vs. 3.2%) were rated as the most important attributes by educators in comparison to students.

**Conclusions:**

No single attribute makes a good health educator; rather health educators are required to have a rounded approach to teaching. Students have greater focus on the educator providing a transfer of knowledge. Educators are additionally focused on professionalism attributes, which may not be valued by students. Students and educators must enter into a clearer understanding of expectations, from both parties, to obtain optimal education outcomes.

## Introduction

Teaching modalities are changing; greater emphasis on blended learning and problem-based learning approaches has seen the role of an educator shift from one who imparts knowledge, to one that facilitates the sourcing, appraising and implementation of knowledge.^1^ Given this shift in the student-educator paradigm, it is important to identify which attributes compromise a good educator. However, this is difficult to define. Knowledge and effective communication skills are an obvious choice, but there is little evidence to demonstrate which attributes are valued by students and educators alike.

The role of an effective educator is multifaceted, incorporating aspects of information provision, role modelling, facilitation, examination, planning and resource development.^2^ Each of these roles may be influenced by the educator’s relationship with students, content expertise and teaching expertise.^2^ Additionally, the personality attributes of the educator may impact the education process – with arrogance, insensitivity and belittling of students negative traits to impact upon the education process.^3^ An educator’s knowledge of the subject matter, or content, has been demonstrated to influence student achievement positively.^4^ Conversely, there is conflicting evidence with respect to the impact of pedagogical training educators have upon their perceived value as an educator and impact upon student performance.^5^

Attributes that are identified with being a good health educator may differ across medical and health contexts.^6^^-^^9^ Buchel et al. identified that medical students felt it important for an educator to respect their autonomy and independence as clinicians and that it was important for educators to practice evidence-based medicine (EBM).^9^ Conversely, surgical residents rate the ability of their educator to be sensitive to their learning needs and provide positive feedback as indicators of teaching excellence.^6^ Nursing students have identified a preference for their educators to be caring, a good communicator and consistent.^10^

The seminal paper by Suktin et al. reviewed published literature from 1909-2006, in a bid to further examine what attributes make a good health teacher.^11^ Three main characteristics were identified. They are physician, teacher and human - with non-cognitive characteristics involving relationships, emotional states and personality types the most dominant.^11^ Knowledge, clinical/technical competence, enthusiasm, positive relationship with students, and communication skills were highlighted as key attributes to being a good clinical teacher.^11^ These attributes, whilst critical to the clinical educator, are less well understood in educators across broader non-clinical health degrees.

Understanding which attributes are akin to the successful health educator in a changing space of health education is of importance, as better teaching should translate into better learning. The majority of literature to date has concentrated on identifying the attributes of effective health educators in the clinical setting (i.e. medicine and nursing). ^11^ Yet, there is limited information to inform how, if at all, such attributes differ amongst health educators in a non-clinical environment.

The aim of this study was to examine which attributes are most valued in health educators, teaching in a non-clinical environment. We specifically wanted to determine whether these perceptions differed between students and educators, and whether these values differed within students.

## Methods

### Study design and participants

A cross-sectional study was conducted with first-year health science students and educators involved with the Bachelor of Health Science degree at Monash University, Australia. Participants were eligible to partake in the study if they were a first-year health science student, or educator teaching into a first-year unit, within the Bachelor of Health Science program at Monash University.

A convenience sample of students and educators was performed. Educators were invited to the study through email invitation. Students were invited to participate in the study by staff at the conclusion of a tutorial. A total of 147 students and 15 educators met the inclusion criteria and were invited to participate in the study. A total of 94 (63.9%) students and 15 educators (100%) responded and completed the questionnaire.

Ethics approval for this project was obtained from the Monash University Human Research Ethics Committee (MUHREC). The project was deemed as low-risk research, as the research involved completing a non-identifiable questionnaire. To minimise any conflict of interest, author DI (the course coordinator) was not involved in the distribution or analysis of data.

### Data collection methods

A previously developed 15 item questionnaire was distributed to students and educators.^9^ The 15 items represented attributes of effective teaching in a medical context. Each teaching attribute also contained a concise definition to ensure clear understanding by all respondents, Appendix 1. The wording of five of the items was amended to reflect teaching practice in the general health sciences. All items were rated on a 5-point Likert scale (1=strongly disagree, 5=strongly agree), with students and educators also asked to rate which three characteristics were viewed as most and least important in a good health educator. 

**Table 1:** Student (n=94) responses on important of attributes of an effective health educator

Attribute	Student response % (n)				
	Strongly disagree	Disagree	Neither disagree or agree	Agree	Strongly agree
1. Enthusiasm	3.2 (3)	1.1 (1)	2.1 (2)	45.7 (43)	47.9 (45)
2. Availability	2.1 (2)	1.1 (1)	17.0 (16)	51.1 (48)	18.7 (27)
3. Clarity	2.1 (2)	2.1 (2)	3.2 (3)	35.1 (33)	57.5 (54)
4. Knowledge base	2.1 (2)	1.1 (1)	2.1 (2)	41.5 (39)	53.2 (50)
5. Feedback skills	2.1 (2)	0 (0)	8.5 (8)	53.2 (50)	36.2 (34)
6. Organisation skills	3.2 (3)	2.1 (2)	6.4 (6)	46.8 (44)	41.5 (39)
7. Professionalism	2.1 (2)	0 (0)	7.5 (7)	47.9 (45)	42.6 (40)
8. Well prepared	2.1 (2)	1.1 (1)	1.1 (1)	47.9 (45)	47.9 (45)
9. Scholarly activity	1.1 (1)	5.4 (5)	48.4 (45)	34.4 (32)	10.8 (10)
10. Non-judgemental	2.2 (2)	1.1 (1)	5.4 (5)	32.3 (30)	59.1 (55)
11. Respects students	2.2 (2)	1.1 (1)	7.5 (7)	44.1 (41)	45.2 (42)
12. Sincerity	2.1 (2)	0 (0)	6.4 (6)	54.3 (51)	37.2 (35)
13. Listening skills	2.2 (2)	0 (0)	5.4 (5)	43.2 (43)	43.2 (43)
14. Practices EBM	2.1 (2)	1.1 (1)	14.9 (14)	55.3 (52)	26.6 (25)
15. Role model	2.1 (2)	2.1 (2)	12.8 (12)	50.0 (47)	33.0 (31)

### Procedure

All participants (students and educators) were provided with a copy of a written questionnaire by a research member at the conclusion of a tutorial. All participants had up to 20 minutes to complete the questionnaire, with all questionnaires (completed or otherwise) provided to the researcher administering the questionnaire and inserted into an envelope, which was sealed until the data transcription process commenced. All data were transcribed from written questionnaire into a digital spreadsheet by a researcher, independent to the study.

### Data analysis

Data analysis was performed independently by two study researchers. Descriptive statistics were generated. We then performed comparisons of the top ranked educator attributes between students and educators, and among male and female students, using Pearson’s chi-square statistics, with z-values reported. The margins of error for the comparisons were obtained by calculating 95% confidence intervals of the difference between the group proportions. A p-value<0.05 was considered statistically significant. Final data analysis was performed using statistical packages SPSS (version 22) and STATA (version 12).

**Table 2:** Comparing the three most and three least important health educator attributes among male and female students

Attribute	3 most important attributes			3 least important attributes		
	Female (n=70)	Male (n=24)	z-value, p-value	Female (n=70)	Male (n=24)	z-value, p-value
1. Enthusiasm	55.7 (39)	25.0 (6)	0.25, 0.01*	5.7 (4)	4.2 (1)	0.28, 0.77
2. Availability	25.7 (18)	25.0 (6)	0.06, 0.94	4.3 (3)	8.3 (2)	-0.75, 0.45
3. Clarity	37.1 (26)	41.7 (10)	-0.40, 0.69	8.6 (6)	4.2 (1)	0.71, 0.48
4. Knowledge base	48.6 (34)	54.2 (13)	-0.47, 0.63	4.3 (3)	0 (0)	1.03, 0.31
5. Feedback skills	21.4 (15)	25.0 (6)	-0.36, 0.71	2.9 (2)	0 (0)	0.84, 0.40
6. Organisation skills	14.3(10)	16.7 (4)	-0.29, 0.77	5.7 (4)	0 (0)	1.20, 0.23
7. Professionalism	2.9 (2)	12.5 (3)	-1.80, 0.07	20.0 (14)	12.5 (3)	0.82, 0.41
8. Well prepared	27.1 (19)	4.2 (1)	2.33, 0.02*	1.4 (1)	12.5 (3)	-2.33, 0.02*
9. Scholarly activity	4.3 (3)	0 (0)	1.03, 0.30	55.7 (39)	54.2 (13)	0.13, 0.89
10. Non-judgemental	7.1 (5)	8.3 (2)	-0.19, 0.84	14.3 (10)	16.7 (4)	-0.28, 0.77
11. Respects students	8.6 (6)	8.3 (2)	0.04, 0.96	14.3 (10)	12.5 (3)	0.22, 0.83
12. Sincerity	10.0 (7)	12.5 (3)	-0.34, 0.73	20.0 (14)	33.3 (8)	-1.32, 0.19
13. Listening skills	2.9 (2)	8.3 (2)	-1.12, 0.26	8.6 (6)	8.3 (2)	0.45, 0.96
14. Practices EBM	4.3 (3)	8.3 (2)	-0.75, 0.45	41.4 (29)	25 (6)	1.43, 0.16
15. Role model	1.4 (1)	8.3 (2)	-1.67, 0.10	55.7 (39)	58.3 (14)	-0.22, 0.83

*Significant at p<0.05

**Table 3:** Comparing the three most and three least important health educator attributes as ranked by students and educators

	3 most important attributes			3 least important attributes		
Attribute	Students % chosen (n/95)	Educators % chosen (n/15)	z-value, p-value	Students % chosen (n/95)	Educators % chosen (n/15)	z-value, p-value
1. Enthusiasm	47.9 (45)	33.3 (5)	1.05, 0.29	5.3 (5)	20.0 (3)	-2.03, 0.04*
2. Availability	25.5 (24)	13.3 (2)	1.31, 0.31	5.3 (5)	13.3 (2)	-1.17, 0.24
3. Clarity	38.3 (36)	20.0 (3)	1.37, 0.17	7.4 (7)	0 (0)	1.08, 0.28
4. Knowledge base	50.0 (47)	13.3 (2)	2.35, 0.01*	3.2 (3)	6.7 (1)	-0.67, 0.51
5. Feedback skills	22.3 (21)	0 (0)	2.03, 0.04*	2.1 (2)	13.3 (2)	-2.16, 0.03*
6. Organisation skills	14.9 (14)	13.3 (2)	0.16, 0.88	4.3 (4)	6.7 (1)	-0.41, 0.68
7. Professionalism	5.3 (5)	20.0 (3)	-2.03, 0.04*	18.1 (17)	6.7 (1)	1.10, 0.27
8. Well prepared	21.3 (20)	40.0 (6)	-1.58, 0.12	4.3 (4)	53.3 (8)	-5.61, 0.00*
9. Scholarly activity	3.2 (3)	20.0 (3)	-2.65, 0.01*	55.3 (52)	0 (0)	3.76, 0.00*
10. Non-judgemental	7.4 (7)	6.7 (1)	0.01, 0.92	14.9 (14)	6.7 (1)	0.85, 0.39
11. Respects students	8.5 (8)	20.0 (3)	-1.37, 0.17	13.8 (13)	13.3 (2)	0.05, 0.96
12. Sincerity	10.6 (10)	6.7 (1)	0.49, 0.64	23.4 (22)	6.7 (1)	1.47, 0.14
13. Listening skills	4.3 (4)	0 (0)	0.81, 0.42	8.5 (8)	13.3 (2)	-0.59, 0.55
14. Practices EBM	5.3 (5)	13.3 (2)	-1.77, 0.24	37.2 (35)	13.3 (2)	1.81, 0.07
15. Role model	3.2 (3)	26.7 (4)	-3.45, 0.00*	56.4(53)	20.0 (3)	2.62, 0.01*

*Significant at p<0.05

## Results

In total, 75.3% of student participants were female, with a mean age of 20.1±4.1 years, whilst 46.7% of educator participants were female, with a mean age of 32.7±9.3 years.

Among students, more than 75% of students agreed or strongly agreed that 14/15 characteristics were important attributes of a good health educator, excluding ‘scholarly activity’ for which only 42% of students felt this was an important health educator attribute, Table 1. Similar, but stronger patterns were observed among educators with more than 90% of educators selecting agree or strongly agree for the same 14/15 characteristics. Only 40% of educators believed scholarly activity to be an important educator attribute.

Among 94 students, there were 252 and 244 responses for the three most and least important educator attributes, respectively. The three top ranked most important health educator attributes according to students were: knowledge base, enthusiasm and clarity with 49.5%, 47.4% and 37.9% of students selecting these characteristics, respectively, Table 1. The three top-ranked least important attributes were role model, scholarly activity and practices EBM with 53%, 52% and 35% of students selecting these characteristics, respectively. When comparing values by gender, male and female students similarly ranked the most and least important attributes, excluding well prepared and enthusiasm which was more likely to be ranked as an important educator attribute among females compared with males, Table 2.

When comparing the most important attributes between students and educators, educators were significantly more likely to elect scholarly activity, professionalism and role model as important characteristics, while students were more likely to elect knowledge base and feedback skills as important attributes, Table 3. For least important attributes, educators were more likely to rank enthusiasm, feedback skills and well prepared as least important attributes, while students were more likely to rank scholarly activity and role model as least important attributes. All other characteristics were similarly ranked between students and educators.

## Discussion

The attributes that underpin a ‘good’ quality health educator are multifaceted.  In this cross-sectional study of health students and educators opinions regarding effective teaching attributes, there were areas of agreement as well as some interesting differences of opinion. Among the top ranked ‘most important attributes’, students and educators similarly rated enthusiasm, availability, and clarity. Interestingly, educators were split in this opinion as enthusiasm was also rated as one of the least important attributes, an area for future enquiry. Whilst these results are based on educators in a non-clinical environment, they support findings from studies that have examined similar attributes in clinical educators.^6^^,^^7^^, ^^9^^,^^11^

Both students and educators in our study also felt strongly that scholarly activity was not an important attribute of good health educator. This finding has previously been reported in other studies, in which the focus has been on the educator as a practising clinician.^9^^,^^12^^,^^13^ In the current environment where educators are encouraged to conduct research and publish, it is sobering to note that such an attribute does not seem to be valued by students or educators – be it from a clinical or pedagogical viewpoint.

In contrast to these areas of agreement, there are several areas where students and educators reported significant levels of disagreement in what constitutes important educator attributes. Namely, students rated knowledge base and feedback skills among the top educator attributes, while educators, interestingly, did not rate these as important attributes. Educators were also more likely to rate professionalism, role model and well preparedness as important attributes while students did not. The finding that educators, but not students, believe being a role model is an important attribute is similar to other studies.^9^^,^^14^ Some gender differences among students were also noted. While male and female students had similar opinions on most educator attributes, female students were more likely to value well prepared and enthusiasm compared with males. Understanding these gender differences is an area for future exploration.

Our study identified the importance of non-cognitive attributes, as shaping what a good health educator may be. Attributes such as enthusiasm, being non-judgemental, sincerity, as examples of non-cognitive attributes, have previously been reported as potential attributes that may be alterable.^11^^,^^15^ The focus to date when training health educators has been on the subject matter and pedagogical frameworks.^16^ Our study lends support to the notion educators who are able to develop their non-cognitive skills can enhance their performance as educators, but also improve the learning experience of students.^15^^,^^17^

Health professionals and researchers are often asked, as content experts, to lead some form of educational activity; be it lectures, small group work or experiential learning. Some may also have many years of experience in teaching the content. Whilst, ‘two out of three ain’t bad’, greater emphasis must be placed on educators being holistic in their approach and ensuring that an effective rapport is established with students to ensure a safe learning environment, and one that promotes a two-way exchange of knowledge and feedback.^2^

This study provides a novel insight comparing the perceptions of students and educators in their rating of attributes associated with being an effective health educator. This was a cross-sectional study of a single first-year health student and educator cohort. Further research is required to examine what differences are apparent in student perception across year levels, by gender, and how these perceptions change as the student cohort progresses during the course. Data gathered was from a single undergraduate health science course; thereby results may not be transferable to other health courses, including graduate-entry courses. Whilst offering novel information about educators’ self-perception of attributes associated with an effective health educator, small numbers preclude widespread generalisation of these results. In addition, a modest response rate from students (63.9%) cannot rule out the possibility of selection bias among the student cohort. Nonetheless, these results are informative for defining important educator attributes, even if among the more engaged students.

## Conclusions

The need for continued professional development is a given for clinicians.^18^ With the creation of education-focussed academic roles, a similar continual professional development agenda to ensure that health educators are meeting these aspiring targets is needed. Our study has identified that aspiring targets should include enthusiasm, availability and clarity. Scholarly activity, though important in a research context, should not be a requirement when building capacity in future health educators. Further research is required to ascertain the apparent disconnect between what attributes students and educators perceive as being important to being a good health educator. This research should examine what influence student maturity has upon perceived importance of attributes of effective health educators, and how this may differ across health disciplines.

### Conflict of Interest

The authors declare that they have no conflict of interest.

## Supplementary material

Supplementary file 1 Appendix 1. Survey instrument: attributes of health educators
