# Supplementary material for: What are the attributes of a good health educator?
Source: Int J Med Educ. 2016 Jun 28;7:206–11. doi: 10.5116/ijme.5765.0b6a (PMC4939217; doi:10.5116/ijme.5765.0b6a)
Supplement: Supplementary file 1 — Appendix 1. Survey instrument: attributes of health educators [file ijme-7-206-S1.pdf]

Appendix 1

Survey instrument: attributes of health educators

Attribute	Description
1. Enthusiasm	Energetic and interested in teaching, positive attitude, enjoys their job, doesn't complain
2. Availability	Easily accessible, willing to come in after hours, answers pages promptly and courteously, allows adequate time for teaching, not hurried or rushed, not distracted
3. Clarity	Answers questions clearly and definitively, summarises teaching points, able to explain difficult topics
4. Knowledge base	Competent across health issues, knows the medical literature, engaged in continuing education
5. Feedback skills	Encourages two-way communication provides timely positive and negative feedback
6. Organisation skills	Efficient, good at time management, respectful of students' time pressures and able to adjust accordingly
7. Professionalism	Respects staff and students; appropriate decorum/dress
8. Well prepared	For lectures, presentations, and other teaching activities
9. Scholarly activity	Active in research, many publications, nationally renowned
10. Non-judgemental	Provides a safe learning environment, non-threatening, does not belittle students, creates an atmosphere wherein students feel safe to admit they don't know the answer
11. Respects students autonomy/independence	Treats students as adult learners does not 'micro' manage
12. Sincerity	Genuine, honest, open, upfront, willing to admit when wrong or doesn't know the answer
13. Listening skills	Listens attentively, does not interrupt, seems interested
14. Practices EBM	Comfortable and confident in the principles and application of evidence-based medicine, knows where to find resources/references for evidence-based medicine (EBM)
15. Role model	Worth emulating in terms of interactions with students, staff, achieves a healthy balance between professional/personal/spiritual/physical life
